# The Effect of Freezing on Non-invasive Prenatal Testing

**DOI:** 10.1038/s41598-019-42980-7

**Published:** 2019-05-06

**Authors:** Xiaolei Xie, Fuguang Li, Weihe Tan, Weiguo Yin, Feiyan Chen, Xiaoyan Guo

**Affiliations:** 1 Prenatal Diagnosis Center, The Sixth Affiliated Hospital of Guangzhou Medical University, Qingyuan People’s Hospital, Qingyuan, Guangdong 511518 China; 2 Molecular Diagnosis Center, The Sixth Affiliated Hospital of Guangzhou Medical University, Qingyuan People’s Hospital, Qingyuan, Guangdong 511518 China; 3 Guangzhou KingMed Company, Guangdong, China

**Keywords:** Genetics, Diseases

## Abstract

Plasma cryopreservation is unavoidable in China, due to technical specifications requiring storage of additional plasma at −80 degrees for three years. However, the effect of freezing on non-invasive prenatal testing (NIPT) is still uncertain. We collected 144 euploid pregnant samples, 22 on trisomy 21, 4 on trisomy 13, and 3 on trisomy 18, by massively parallel sequencing before and after freezing. Compared with the success rate of 100% of fresh samples, the detection success rates of trisomy 21, trisomy 13 and euploidy in frozen samples by NIPT were 95.45%, 75% and 95.14%, respectively. Of these, 9 cases of frozen sample sequencing failed, with 8 cases being due to high GC content. The chromosome 21 (chr21) z-value of the frozen trisomy 21 samples was lower than that of fresh samples. Meanwhile, freezing reduced the male positive foetal cell-free DNA (cfDNA) fraction, which was accompanied by an increase in the Unimap-GC level in the massively parallel sequencing data and a decrease in the Unique reads/Total reads ratio. Laboratory freezing reduced the chr21 z-value of foetal trisomy 21, which can be explained by a reduction in the foetal cfDNA fraction and effective Unique reads for NIPT analysis. The Unimap-GC content of the serum samples after freezing was higher, which can lead to failure of NIPT detection.

## Introduction

Chromosomal foetal aneuploidies represent a major class of genetic defects, including trisomy 21, trisomy 18, trisomy 13 and sex chromosome aneuploidies^1,2^. Most notably, the incidence and hazard of trisomy 21 (Down syndrome) for families are much higher compared to trisomy 18 and 13, with the current occurrence rate for trisomy 21 being one in 417 newborns^2^. Traditional serum screening is not effective for trisomy 21 detection^3^. With the development of next generation sequencing technology, non-invasive prenatal testing (NIPT) for foetal chromosomal aneuploidy serum screening has been validated by various clinical trials, and NIPT has been proven to be highly sensitive and specific for trisomy 21^4–6^.

Foetal cell-free DNA (cfDNA) is found in maternal plasma and serum^7^. One important clinical application is the use of cfDNA in maternal blood for non-invasive prenatal diagnosis^8,9^. Foetal trisomy determination of specific chromosomes depends on a small increase in the proportion of the chromosome and is detected by a z-test. The foetal cfDNA fraction is an important parameter to determine the z value for foetal chromosomal aneuploid assessment^10,11^.

During the NIPT laboratory testing process, the remaining maternal plasma samples are often stored at −80 °C for a long period of time. However, the effect of freezing on NIPT detection is still uncertain. In this study, we investigated the impact of freezing on the performance of NIPT testing, thus allowing us to understand the effect of laboratory treatment on trisomy 21 detection sensitivity.

## Results

### Maternal plasma DNA sequencing before and after freezing

Maternal characteristics of the study population are presented in Table 1. We collected 144 euploid pregnant samples, 22 on trisomy 21, 4 on trisomy 13, and 3 on trisomy 18 for non-invasive prenatal cell-free DNA sequencing before and after freezing. The median gestational week for trisomy 21 was 17 (range 12–23), which is slightly older than the trisomy13 and 18 groups. In the clinical routine, traditional serum screening abnormalities would be required for NIPT, so the T21 gestational week was slightly older. Trisomies 13 and 18 were often accompanied by malformations that can be detected by B-ultrasound, suggesting early detection of gestational week. The gestational age of all samples was between 12 and 25 weeks. The freezing time of each group was different, with the longest freezing time in the trisomy 13 group, with a median of 76 days, and the shortest freezing time in the euploid group, with a median of 13 days.

**Table 1:** Characteristics of pregnant women within the study population.

	n	Median, range		
		Maternal age (years)	Gestational weeks	Freezing time (days)
Trisomy 21	22	38.5 (15.0–44.0)	17.0 (12.0–23.0)	25.0 (12.0–88.0)
Trisomy 13	4	30.0 (30.0–40.0)	16.0 (16.0–20.0)	76.0 (14.0–78.0)
Trisomy 18	3	36.0 (35.0–41.0)	16.0 (15.0–20.0)	44.0 (38.0–45.0)
Euploid	144	31.0 (16.0–43.0)	17.0 (12.0–25.0)	13.0 (9.0–18.0)

Among the 173 samples, all maternal plasma DNA first sequenced without freezing was successful. The foetal chromosomal aneuploidy or euploidy, including trisomy 21, trisomy 18, and trisomy 13, was accurately identified with a detection sensitivity of 100% and a detection specificity of 100%. After freezing serum samples at −80 °C, the detection success rates of trisomy 21, trisomy 13 and euploidy on NIPT were 95.45%, 75% and 95.14%, respectively. Nine cases of plasma cfDNA for a second sequencing failed, including 1 case of trisomy 21, 1 case of trisomy 13, and 7 cases of euploidy. The median maternal age and gestational weeks for the nine cases in which sequencing failed were 34.5(range 27–44) and 16 (range 13–25), respectively. There was no specific distribution of maternal age or gestational week in these 9 failed cases. Among these cases, 8 cases were due to high GC content, and 1 case of trisomy 13 detection failed. All failed results were interpreted as redo by the BambniTest software. Details are shown in Table 2.

**Table 2:** Summary of the syndromes detected before and after freezing.

Syndrome	The detection success ratios of NIPT	
	Fresh samples	Frozen samples
Trisomy 21	100% (22/22)	95.45% (21/22)
Trisomy 13	100% (4/4)	75% (3/4)
Trisomy 18	100% (3/3)	100% (3/3)
Euploid	100% (144/144)	95.14% (137/144)

### Freezing reduces the z value of trisomy 21

**Figure 1:**
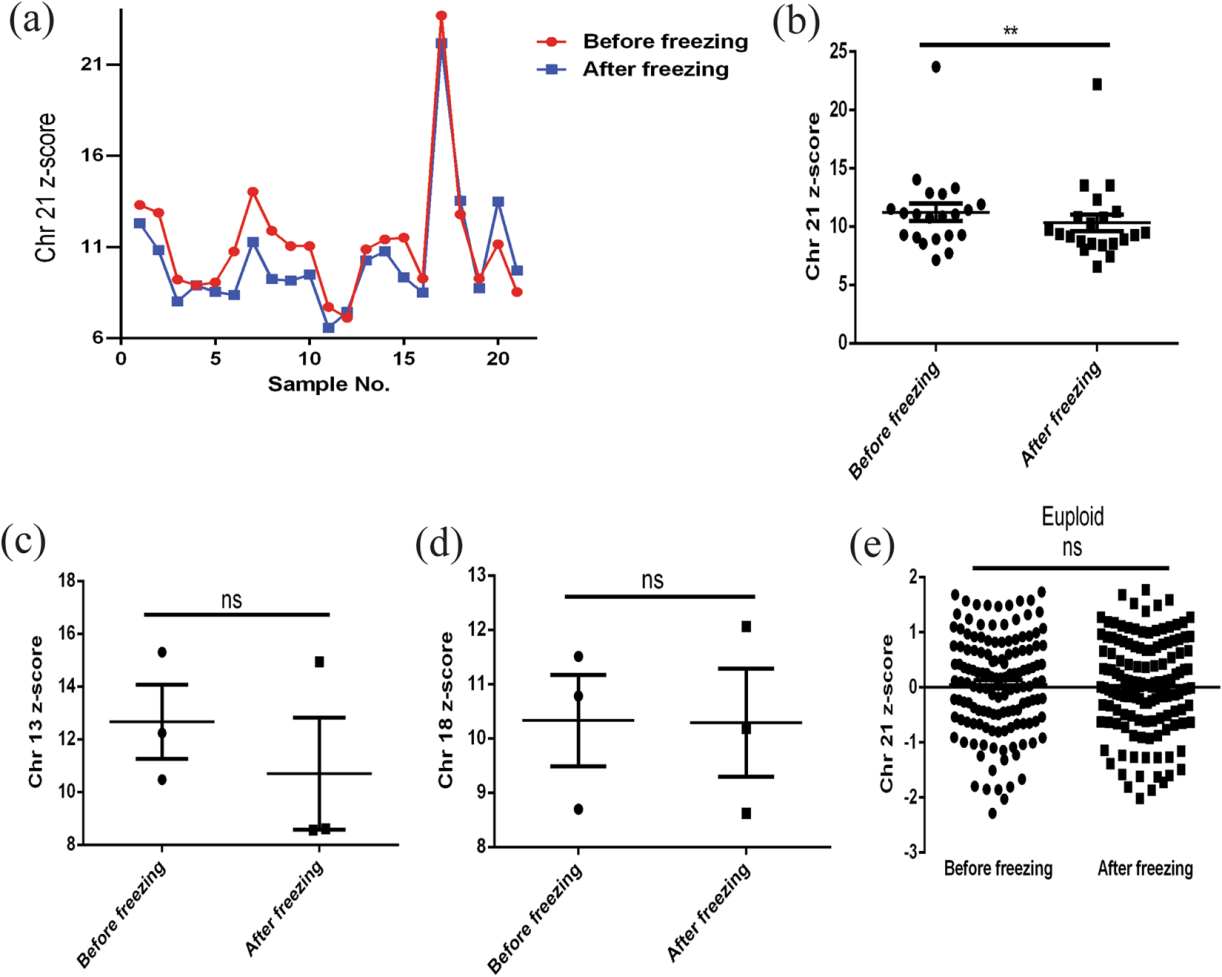
Non-invasive prenatal testing of trisomy 21,13,18 or euploid foetus samples before and after freezing. (**a**,**b**) Comparison of the chr21 z-score of trisomy 21 before and after freezing. (**c**) Comparison of the chr13 z-score of trisomy 13 before and after freezing. (**d**) Comparison of the chr18 z-score of trisomy 18 before and after freezing. (**e**) Comparison of the chr21 z-score of the euploid foetus before and after freezing. Error bars are the SEM. **P < 0.01, paired *t* test.

The results of NIPT were consistent before and after freezing except for the detection failure of a few samples. Comparing the chromosome 21 (chr21) z value of NIPT between fresh samples and frozen samples, 80.95% (17/21) of fresh sample values were higher than frozen values, and 19.05% (4/21) of the values were lower than frozen values (Fig. 1a). Our data show that the chr21 z values of the fresh sample group were significantly higher than the values of the frozen group by using a paired *t* test (Fig. 1b). There was no significant difference between the chromosome 13 (chr13) z value and the chromosome 18 (chr18) z value before and after freezing (Fig. 1c,d). The normal euploid foetus exhibited no obvious difference in fresh and frozen samples (Fig. 1e).

### Comparing the sequencing data before and after freezing

**Figure 2:**
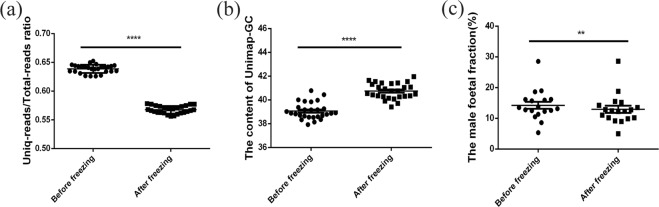
Analysis of the sequencing data on trisomy 21,13 or 18 samples before and after freezing. (**a**) Comparison of the ratios of Uniq-reads/Total-reads in 27 positive samples, including 21 trisomy 21, 3 trisomy 13and 3 trisomy 18. (**b**) Comparison of the content of Unimap-GC in 27 positive samples before and after freezing. (**c**) Comparison of the positive male foetal fraction before and after freezing. Error bars are the SEM. **P < 0.01, ****P < 0.0001, paired *t* test.

We next investigated the sequencing data of 27 positive samples (21 trisomy 21, 3 trisomy13 and 3 trisomy18) before and after freezing. There were no significant differences in total reads and uniq-reads between the two groups (see Supplemental Fig. 1), but the ratios of Uniq-reads/Total-reads in fresh samples were higher than the ratios in frozen samples (Fig. 2a). This suggests that more effective sequencing data in fresh samples were used to evaluate the NIPT results. At the same time, the content of Unimap-GC upon freezing increased significantly (Fig. 2b).

To confirm whether changes in foetal cfDNA fractions occur due to freezing, we further selected and compared the foetal fraction in 18 cases of male positive samples (14 trisomy 21, 2 trisomy13 and 2 trisomy18). The minimum chromosome Y (chrY) z-value of male positive samples was 22.47, and the foetuses were confirmed to be male by follow-up. The result showed that the foetal cfDNA fraction in fresh samples was slightly higher than the fractions in frozen samples by using the paired *t* test (Fig. 2c).

## Discussion

Non-invasive prenatal testing by massively parallel sequencing has been reported since 2008^12^. Numerous studies proved that NIPT was highly accurate for detecting foetal chromosomal aneuploidies^2,5,6,8^, which presents a new era of prenatal screening. Frozen serum samples are unavoidable in the process of NIPT detection in the laboratory. The findings in this study demonstrated that the chr21 z-value of NIPT detection was reduced by laboratory freezing treatment. Meanwhile, freezing reduced the male foetal cfDNA fraction. It was also shown that the content of Unimap-GC in frozen samples increased, whereas the Unique reads/Total reads ratio decreased.

Foetal cfDNA is a key determinant that affects the performance of foetal DNA-based prenatal tests^11,13^. The foetal fraction is derived from apoptotic trophoblastic cells in the placenta^14^. Samples with sufficient foetal fractions that pass quality control metrics can provide an accurate assessment of the chromosomes tested. A variety of methods are used to calculate foetal DNA concentration^15,16^. In our study, freezing led to a decrease in foetal DNA concentration after cryopreservation. With the reduction of effective sequencing data for NIPT analysis, it is reasonable to speculate that the chr21 z-value decreases after freezing. Further analysis revealed that the 21-trisomy foetal cfDNA concentration did not decrease with the freezing time, suggesting that freezing may affect the chr21 z-value regardless of the length of freezing time. This reminds physicians and examiner to avoid freezing the NIPT plasma during transport or storage, especially plasma with low foetal cfDNA concentrations. The freezing time was different among groups. Trisomy 13 was frozen the longest and showed the worst performance. Due to limitation of the number of samples, we cannot make an inference regarding whether the long freezing time leads to a low detection success rate.

On average, the amount of foetal cfDNA in plasma from a pregnant woman is approximately 2–20%, but there is a large variance in the fraction of foetal cfDNA between patients^17^. The higher the percentage of foetal cfDNA, the more effective NIPT is at distinguishing foetal trisomy from a euploid foetus, especially for trisomy 21^16^. This study suggests that the high level of foetal cfDNA concentration and more effective data will increase the detection success rate of trisomy 21, especially the z-value in cut-off regions. It has been speculated that the foetal fraction should be at least 4% to allow for reliable detection of common trisomies^18^. Currently, the threshold we use for accurate detection of trisomy using Illumina’s NextSeq CN500 is a foetal cfDNA fraction of no less than 3.5%. In the 9 cases of NIPT detection failure after freezing, no sample was detected due to the cfDNA concentration being below 3.5%.

Foetal cfDNA is affected by many factors, including the gestational age^19^, maternal weight^16^ and physical activity^20^. The cfDNA in plasma increases as pregnancy progresses, but it decreases with maternal weight gain. There seemed to be a slight positive correlation between the foetal DNA fraction and the crown-rump length (CRL), log freeβ-hCG MoM and log PAPP-A MoM^16^. However, the nuchal translucency (NT) and serum prenatal screening results in the high-risk and low-risk groups showed no significant differences in the foetal cfDNA fraction^21^. In the samples positive for trisomies 13 and 18, the foetal fractions were significantly lower compared with that of the NIPT-negative cases and trisomy21, indicating that different foetal aneuploidies have varied effects on the foetal DNA fraction^22,23^.The higher foetus fractions in trisomy 21 may be one of the reasons that the detection performance for trisomy 21 was better than that of trisomy 13 or 18. These studies indicate that trisomy 21 is more susceptible to foetal fraction. Because of the limited number of trisomies 13 and 18, we did not see the effect of the foetal fraction changes in trisomies 13 and 18 after freezing in this study. Future research should be examined whether increased GC content in cfDNA after cryopreservation means that freezing more likely destroys the two hydrogen bonds between adenine and thymine, resulting in an increased GC content.

In conclusion, this study determined that freezing decreased the chr21 z-value of NIPT detection. Laboratory freezing reduced the foetal cfDNA concentration, accompanied by an increase in the Unimap-GC content in the massively parallel sequencing data and a decrease in the Unique reads/Total reads ratio. The findings in this study provide one laboratory factor affecting the accuracy of NIPT detection, especially for trisomy 21, which will help to further understand cfDNA characteristics.

## Materials and Methods

### Study design and sample collection

Samples from 173 pregnant women were collected between April 2017 and July 2017 for this study. The research adhered to the tenets of the Declaration of Helsinki on research involving human subjects. The study design was approved by the ethics committee of the Six Affiliated Hospital of Guangzhou Medical University. All participants gave informed written consent. The pregnant women were enrolled as participants and were determined to be high risks for aneuploidy by conventional serum screening, single serum index abnormalities, abnormal foetal ultrasound or maternal age (≥35 years). All participants (range 12 to 25 weeks gestation) required NIPT detection to avoid foetal trisomies 21,18 and 13. One hundred seventy-three pregnant women bearing 99 male foetuses and 74 female fetuses participated in the study. Follow-up information was acquired by telephone. All positive results of NIPT detection were confirmed by prenatal diagnosis for chromosome karyotype analysis. The foetus gender was confirmed by follow-up after birth. Ten milliliters of maternal peripheral blood was collected into cell-free DNA BCT tubes (Streck company, La Vista, USA) for NIPT detection.

### Maternal plasma processing, cfDNA extraction and sequencing

The peripheral maternal blood samples were sent to a clinical laboratory within 2 hours. The blood samples were centrifuged first at 1,600 *g* for 10 min at 4 °C. The plasma was transferred to microcentrifuge tubes and then centrifuged again at 16,000 *g* for 10 min to remove residual cells. Each blood sample was separated into three tubes of plasma (1.2 ml serum/tube), with the fresh first tube used for a follow-up experiment and the rest stored at −80 °C. On the first day, we extracted cfDNA in 1.2 ml of serum from pregnant women using the Berry Genomics Nucleic Acid kit (Berry genomics company, Beijing, China). The cfDNA was dissolved in 40 μL of Tris-HCL solution (10 mM), with a concentration greater than 0.5 ng/μL. The next day, all cfDNA was used as the input DNA to create a library using the library prep kit from Berry Genomics. Plasma DNA libraries of 95 samples were indexed using 6 bp indexing oligos and quantitated by the SYBR fast qPCR kit from Kapa Biosystems (Woburn, MA, USA). The library concentration of each sample must be greater than 10pM before sequencing. On the third day, each sample library was pooled and loaded into a NextSeq flow cell at 3pM DNA concentration. Clustering and sequencing were conducted according to Illumina’s NextSeq CN500 instructions using the single-ended 45 bp sequencing protocol. The time of the NIPT clinical reports for the fresh samples was no more than 7 working days. The procedure of NIPT detection in frozen samples was the same as in fresh samples.

### Sequencing data analysis

A analysis of sequencing reads was performed by the BambniTest software (Berry genomics company, Beijing, China). Sequencing reads were mapped to the non repeat-masked reference human genome (hg18) using the SOAP bioinformatic algorithm^24^. Unique reads were the only mapped one chromosome DNA sequences, which were analysed for follow-up counting. The number of unique sequence reads aligned to each chromosome was counted, and the Unimap-GC content was calculated. To offset the GC bias generated between runs, the final displayed Unimap-GC was corrected by the BambniTest^25^. If the Unimap-GC content was ≥42%, the software will interpret the sample as having high GC content. High GC content and sequencing detection failure were all defined as redo by the BambniTest software.

For each chromosome of each sample, the unique chromosome representation (% chrN) value was generated according to the following equation: % chrN = Unique count for chrN /Total unique count of the sequences mapped to all the autosomes. The assessment of foetal aneuploidy risk was according to the formula chrN z-score = (% chrN- mean% chrN_reference_)/S.D.% chrN_reference_. The cut-off z-value was 3^12^. Four hundred twelve pregnant women with euploidy foetuses were selected as the reference set. The mean (% chrN_reference_) and the standard deviation (% chrN_reference_) of the reference set were used for calculating z-scores. In this sequencing platform, the lowest foetal concentration for distinguishing trisomy effectively was 3.5%. Samples below this quality control standard were recommended to re-collect blood or abandon testing.

### Foetal fraction calculation

The male foetal DNA fraction calculation based on unique read counts on the Y chromosome were reliable^15^. We calculated the proportion of male foetal DNA based on the calculation method published by Irena H *et al*.^16^.

The male foetal DNA fraction = (% chrY-female % chrY)/(male % chrY- female % chrY), where % chrY represents the ratio of unique reads of the Y chromosome to the number of unique reads on all chromosomes; female % chrY is the average of % chrY in 200 pregnant women bearing euploid female foetuses; and male % chrY represents the average of the ratio of % chrY of two adult male volunteers.

### Statistical analysis

Data were analysed using GraphPad Prism 6.0 software. Data between two groups were analysed by a two-tailed paired *t* test. Values are presented as the mean ± SEM. Statistical significance was set at **P < 0.01 and ****P < 0.0001.

## Supplementary information

Supplemental Figure-1

## Data Availability

The data generated during and/or analysed during the current study are available from the corresponding author by reasonable request.
