# Supplementary material for: The Effect of Freezing on Non-invasive Prenatal Testing
Source: Sci Rep. 2019 May 6;9:6962. doi: 10.1038/s41598-019-42980-7 (PMC6502861; doi:10.1038/s41598-019-42980-7)

**The Effect of Freezing on Non-invasive Prenatal Testing**

Xiaolei Xie^1,2^, Fuguang Li^1^, Weihe Tan^1^, Weiguo Yin^2^, Feiyan Chen^3^ and Xiaoyan Guo^1^*

^1^Prenatal Diagnosis Center, ^2^Molecular Diagnosis Center, The Sixth Affiliated Hospital of Guangzhou Medical University, Qingyuan People’s Hospital, Qingyuan, Guangdong 511518, China; ^3^Guangzhou KingMed Company, Guangdong, China

Supplemental Figure 1

Analysis of the sequencing data on trisomy 21,13 or 18 samples before and after freezing. (a) Comparison of the amounts of total-reads in 27 positive samples, including 21 trisomy 21, 3 trisomy 13 and 3 trisomy 18. (b) Comparison of the amounts of Uniq-reads in 27 positive samples before and after freezing.


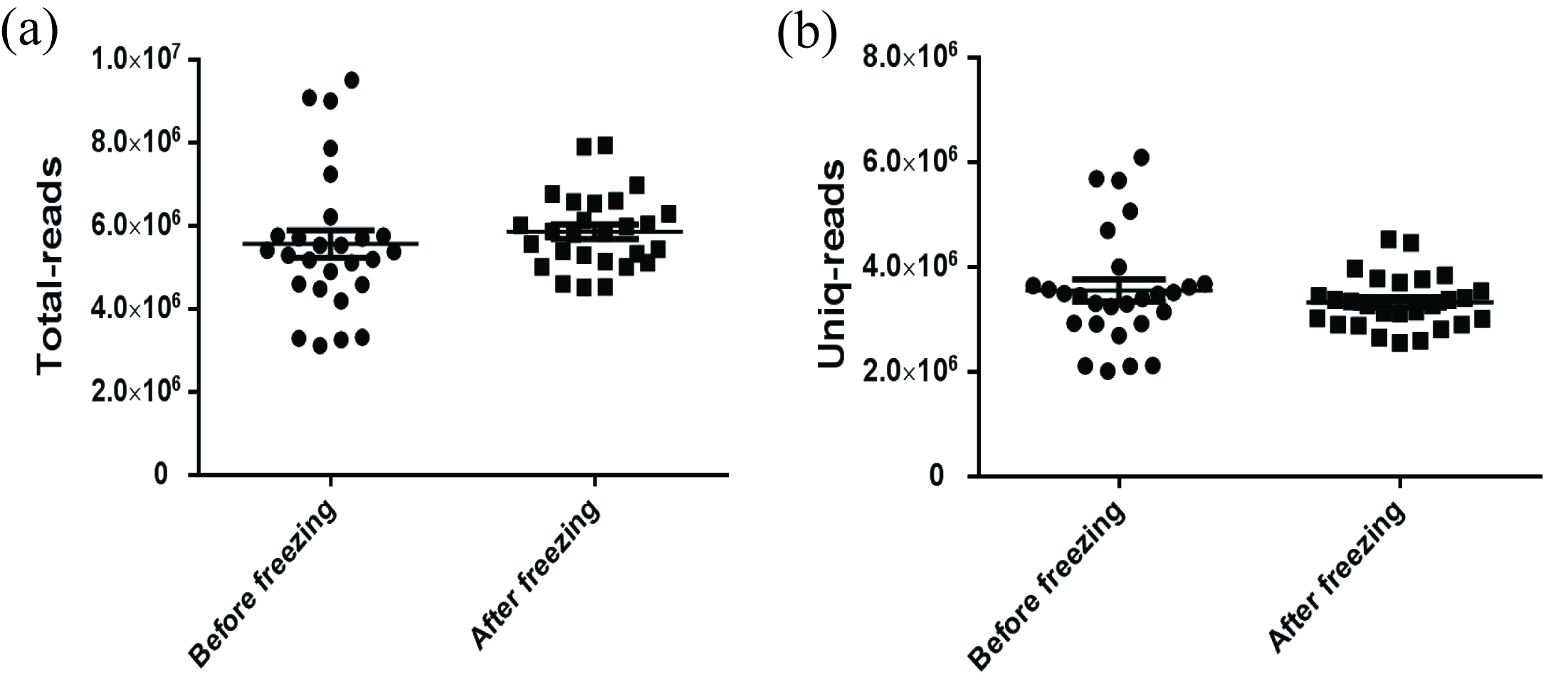

Supplement: Supplementary file 1 — Supplemental Figure-1 [file 41598_2019_42980_MOESM1_ESM.docx]
